# Semiology of seizures with temporo‐polar or “medio‐lateral” temporal origin: A systematic review

**DOI:** 10.1002/epd2.20329

**Published:** 2025-03-12

**Authors:** Andreas Schulze‐Bonhage, Victoria San Antonio‐Arce

**Affiliations:** ^1^ Freiburg Epilepsy Center, Member of the ERN EpiCARE, Faculty of Medicine University of Freiburg Freiburg Germany

**Keywords:** anatomo‐clinical correlation, epilepsy surgery, focal epilepsy, ictal semiology, lateral temporal lobe, mesial temporal lobe, systematic review, temporal pole

## Abstract

A systematic review using PRISMA criteria was used to review the literature regarding the specific semiology of seizure arising (a) from the temporal pole or (b) from both medial and lateral temporal cortex. Evidence was analyzed with regard to information provided by intracranial EEG recordings and surgical outcomes, and an estimation of validity of reported signs and symptoms was performed. Semiology of seizures originating from the temporal pole was mostly related to diverse patterns of ictal spread rather than to the localization of seizure origin and comprised a wide variety of early signs and symptoms. Seizures with rapid involvement of temporo‐medial and temporo‐lateral cortex were intermediate in semiology between medial and lateral onset seizures and may have more frequently early automatisms and early vocalization than seizures arising from temporo‐medial or temporo‐lateral cortex only. Results of this review are discussed as to limiting factors of origin‐based analyses for the understanding of seizure semiology.

**Key points**

- The semiology of seizures of temporo‐polar origin is highly variable depending on seizure propagation to frontal, insular or temporal regions.
- Seizures with rapid propagation from mesial to lateral temporal regions or vice versa have characteristics intermediate between temporomesial and temporalateral origin, they can have early automatisms and vocalizations.

## 1 INTRODUCTION

Whereas the International League against Epilepsy (ILAE) distinguishes mesial temporal lobe epilepsy and neocortical temporal lobe epilepsy, functional specialization and connectivity differ in temporal subregions. Depending on the eloquence of these areas and on potentially different spread or sequence of spread to other brain areas, this may lead to differences in the semiological appearance of seizures of temporal lobe subregions. Primary and secondary auditory cortices and the temporobasal language area are examples of eloquent regions within the temporal lobe which show characteristic primary symptoms when involved during seizures like auditory hallucinations or language disturbances. For the temporal subregions of the temporal pole and for seizures called “medio‐lateral” in some French literature^1^, with very early involvement of mesial and lateral parts of the temporal lobe, it is less clear if initial ictal involvement does lead to a characteristic semiological correlate.^2^

Here, a systematic review based on publications reporting the specific semiology of seizures arising from the temporo‐polar and “mesio‐lateral” temporal cortex is given, using PRISMA criteria. Results are discussed with regard to the question if semiology significantly differs from other temporal subregions and if this justifies a functional separation of these subregions in the context of epilepsy, for example, to interpret seizures with regard to their localizing value for presurgical diagnostics, epilepsy surgery, or for local approaches of neurostimulation.^3^, ^4^, ^5^, ^6^, ^7^

## 2 METHODS

To identify publications addressing seizure semiology in relation to temporo‐polar or mesio‐lateral seizure origin, a standardized literature research was performed on the PubMed database using the search string “temporal AND (temporo‐polar OR polar OR mesio‐lateral OR medio‐lateral OR mesiolateral OR mediolateral) AND (seizure* OR epilep* OR semiolog* OR sign*) AND (surg* OR EEG) [human subjects]” according to suggestions of the research group. Results were analyzed by two board‐certified epileptologists with expertise in focal epilepsy in adults (ASB) and children (VSA).

Abstracts were screened with separate evaluation by both authors. Any article with an abstract which pointed to semiological information from either subregions (33/239) was completely studied and analyzed. Seven articles remained which contained semiological information in sufficient granularity; citations within these articles led to the additional integration of 11 publications with semiological information which were not identified by the search string but were integrated into the analyses.

**FIGURE 1:**
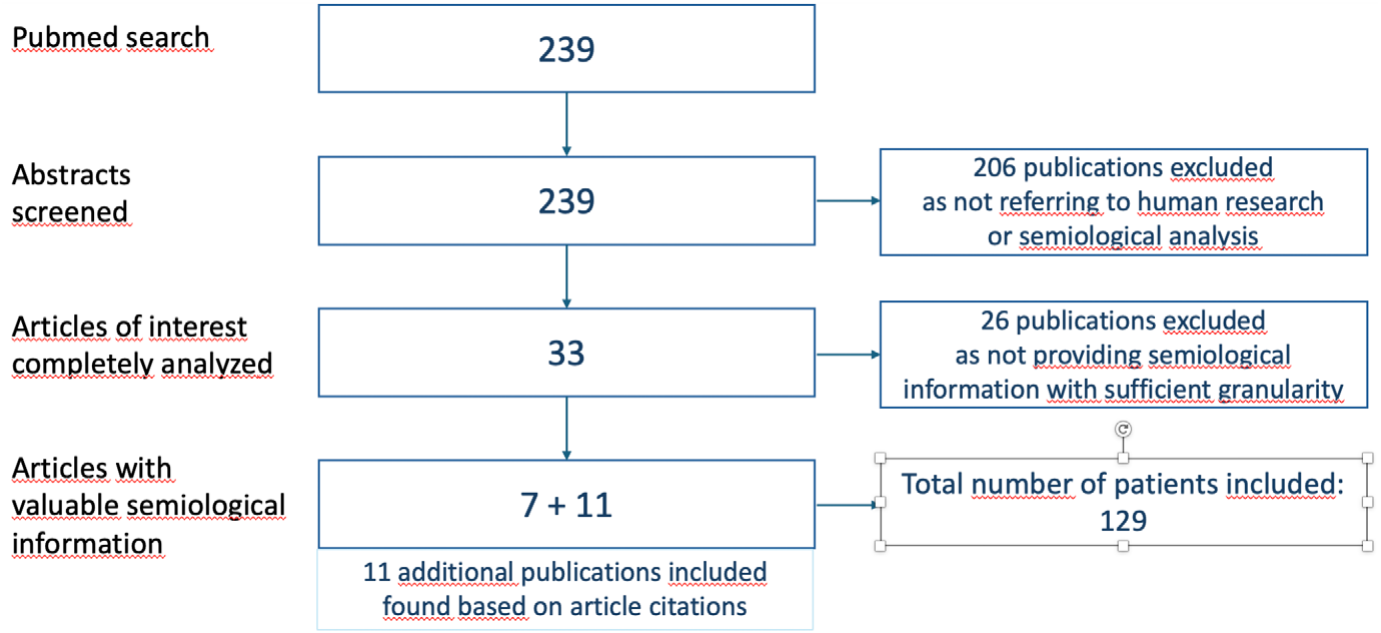
PRISMA scheme of the standardized literature research.

Given the low number of articles on the topic published, there was no exclusion of publications with low patient numbers, that is, also single case reports were included. This resulted in a total of 129 patients in whom semiological data were available with reported temporal pole seizure origin (78/129) or “mesio‐lateral” temporal origin (51/120) (Figure 1).

Studies were analyzed as to the degree of evidence for the localization of seizure origin and to differentiation of symptoms and signs reported as compared to other temporal regions. Selection bias and assessment bias were assessed using QUADAS2 method.^8^ In each of the studies, we analyzed how frequently a symptom was described in the region of interest, and taking into account the risk of inclusion bias and assessment bias, how was the reliability of the reference standard, that is, the confidence in the epileptogenic zone (low, moderate, high, or very high). We only rated with very high confidence the percentage of cases in which the clinical signs were proven to be related to a location by selective surgery resulting in seizure freedom, regardless of whether they had iEEG or not. We rated with high confidence the percentage of cases in which the clinical signs had been proven to be related to a location by iEEG. By contrast, we rated with moderate confidence the cases in which non‐selective surgery or a surgery for which we have no data as to whether or not it was selective has resulted in seizure freedom, and with low confidence, the cases that did not have surgery or were not seizure‐free after surgery, or for which outcome data are not available.

## 3 RESULTS

### 3.1 Seizures of temporo‐polar origin

**FIGURE 2:**
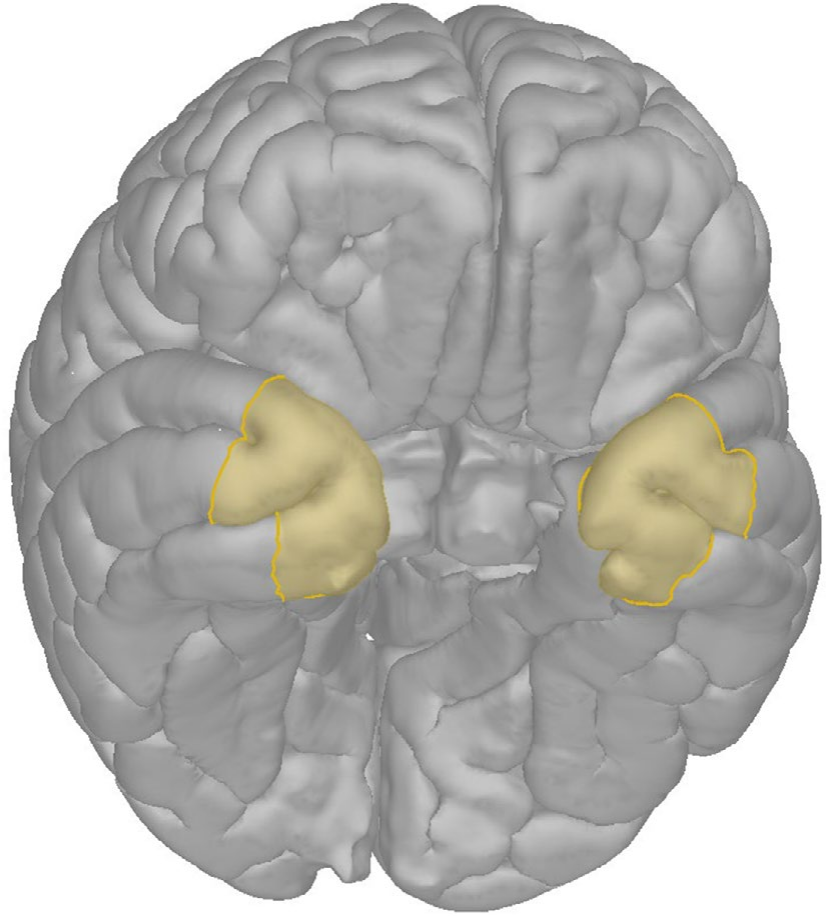
Temporal pole as segmented based on 36 MRI scans according to Fischl et al. ^12^ Note that there are no limiting lateral or basal sulci delineating the pole but rather the anterior end of the superior lateral sulcus as a landmark for segmentation.

The temporal pole is the rostral part of the temporal lobe. As there are no separating sulcus or other landmark on either of the aspects of the temporal lobe, it has been defined as the region anterior to the end of the middle temporal sulcus on the lateral side and anterior to the end of the inferior temporal sulcus on the basal side, with a posterior border corresponding to a line drawn through the anterior edge of the temporofrontal transitional fold (Chabardès et al.^9^, ^10^; Figure 2), yet variable modifications of border definitions have been in use over time (e.g.,^11^).

Serving as a multisensory association area involved in a wide spectrum of cognitive tasks,^13^ it has a wide connectivity with afferents from the hippocampus, entorhinal cortex, occipital cortex via the inferior longitudinal fascicle^14^, ^15^ and furthermore connections with the frontal and insular lobes.^16^, ^17^ Efferents also include the basolateral nucleus of the amygdala, hippocampus, parahippocampal gyrus,^18^, ^19^ and superior temporal gyrus, but also to the orbitofrontal cortex via the fasciculus uncinatus and the longitudinal insular gyri.^20^, ^21^

For discussing evidence for a distinguishable semiology of temporo‐polar seizures as opposed to semiological seizures arising from other temporal regions, evidence related to the epileptological outcome of temporal resections can be obtained only in patients undergoing selective polar resections, leaving particularly the hippocampus and posterior temporolateral and temporobasal regions intact. In recent years, such tailored surgical resections have been increasingly reported either with patients with temporopolar cortical dysplasia 22, 23 or with temporopolar encephaloceles,^24^, ^25^, ^26^, ^27^, ^28^, ^29^ with the latter contributing most to the available high‐level evidence of semiology as based on both intracranial EEG recordings and postoperative outcome. In contrast, many studies report postoperative outcomes following standard anterior temporal lobe resections or at least inclusion of the hippocampus in resections performed (e.g.,^25^, ^28^, ^29^, ^30^, ^31^, ^32^), precluding additional evidence for a seizure origin in a temporal subregion. Furthermore, intracranial coverage of the temporal pole poses particular challenges when using subdural electrodes^33^ but also for SEEG, when performed with an orthogonal approach; this does anatomically preclude coverage of the true polar region and rather provides recordings from temporal areas up the level of the amygdala.

The first study reporting detailed seizure semiology with high evidence for a temporo‐polar onset was from Montreal, reporting the intraoperative detection of temporo‐polar encephalozeles in the pre‐MRI era and seizure‐free outcome in two patients resulting from circumscribed resections.^27^ As seen in Table 1, overall 11 publications were identified for the analysis with a maximum of 23 patients' semiology in seizures with temporo‐polar origin, including three single case reports; the derived evidence is given in Table 2.

**TABLE 1:** Studies identified with the search strategy reporting detailed semiology from the temporo‐polar region.

# study	# patients	Adult (A), children (P)	% MRI positive	% intracranial EEG (SEEG unless stated otherwise)	% operated	% class I ≥ 1 year	Confidence in the EZ (% very high/high/moderate/low)
1	23	23 A	100	0	52 (30% selective polar)	57^a^	17/0/22/61
2	22	22 A	100	18	23 (9% selective)	100^a^	9/9/82/0
3	9	9 A	100	22 (subdural)	66 (22% selective)	100^a^	22/22/0/56
4	8	8 A	100	25 (subdural)	100 (25% selective)	75 (unclear if selective)	0/25/50/25
5	4	4 A	100	100	100 (selective)	75	75/0/0/25
6	3	2 A, 1 P	– (100 other imaging +)	0	100 (66% selective)	50^a^ (50% unknown)	33/0/33/33
7	3	3 A	– (100 other imaging +)	0	100 (all unselective)	n.a.	0/0/100/0
8	3	3 A	100	0	100	100	100/0/0/0
9	1	1 P	100	100 (subdural)	100 (unselective)	n.a.	0/0/100/0
10	1	1 A	100	100	100 (selective)	100	100/0/0/0
11	1	1 A	100	0	100 (selective)	100	100/0/0/0

*Note*: Very high confidence in the epileptogenic zone localized in the temporal pole only was only attributed when selective surgery controlled epilepsy. Studies: 1: Saavalainen et al.^28^; 2: Toledano et al.^29^; 3: Panov et al.^31^; 4: Wang et al.^32^; 5: Fong et al.^34^; 6: Hyson et al.^27^; 7: Leblanc et al.^30^; 8: Abou‐Hamden et al.^24^; 9: Aquilina et al.^25^; 10: de Souza et al.^26^; 11: Martínez‐Pías et al.^35^

^a^ Seizure freedom after selective surgery.

**TABLE 2:** Summary of evidence for a temporo‐polar seizure origin.

	# patients	% adults	% MRI positive	% SEEG	% selectively operated	% of selectively operated class I ≥1 year	High or very high confidence in the EZ
Min.	1	0	(100)**CT‐positive	0	23	39	0
Max.	23	100	100	100	100	100	100
Median	3	100	100	18	100	100	100
Total	78	97	100	28	31	71	28

As pointed out, limited SEEG coverage of the temporal pole and unselective surgery lead to an overall moderate confidence in the epileptogenic zone across the cohorts reported. Nevertheless, there is concordance in all studies reporting several patients there is high interindividual variability of seizure semiologies. This is reflected in the fact that the most frequently reported sign, epigastric auras, was found in only 22% of the patients reported (Table 3).

**TABLE 3:** Anatomico‐clinical correlations in seizures with temporo‐polar origin.

Anatomico‐clinical correlation temporal pole	% reported (min–max)	More at onset or during propagation	Overall grade level (high, moderate, low) that the sign/symptom is associated with the brain region
Epigastric aura^b^	22% (0–43)	At clinical onset	Low
Aura^b^ of fear	13% (0–100)	At clinical onset	Low
Déjà vu	12% (0–100)	At clinical onset	Low
Cephalic aura	4% (0–14)	At clinical onset	Low
Hypermotor^b^	6% (0–50)	At clinical onset or later^a^	Low
Version	6% (0–100)	Later^a^	Low
Dystonic	19% (0–100)	Later^a^	Low
…. Multiple	<5%		Low

^a^ “Later” is used rather than “during propagation” as all signs are considered to occur only after propagation to symptomatogenic areas.

^b^ The term “hypermotor” from the identified publication as well as “aura” is used in this table rather than the present terminology which would be “hyperkinetic” and “focal aware emotional,” etc.

The full spectrum of initial signs reported in the identified literature additionally included a somatosensory aura, other psychic auras including a feeling of pleasure, auditory and cephalic aura, tunnel vision, out‐of‐body experience, nausea, behavioral arrest/staring, grimacing and odd laughter. None of the signs reported in focal epilepsy of temporo‐polar onset has been shown to be more frequent than in seizures with origin from other temporal subregions.

### 3.2 “Medio‐lateral” or “lateral‐medial” seizure origin

The terms “medio‐lateral” and “lateral‐medial” to characterize the seizure onset zones in the temporal lobe appears self‐contradictory at first glance. They were introduced based on Stereo‐EEG recordings using an orthogonal approach by the Marseille group subclassifying seizures based on the speed of propagation from temporo‐lateral to temporo‐mesial recording sites or to the inability of SEEG to distinguish a temporo‐mesial or lateral origin. In the publications from Bartolomei et al.^36^ and of Maillard et al.,^38^ patients constituting this group had delays between the appearance of ictal patterns in temporolateral and temporomesial contacts of 0–3 s; the mesio‐lateral group was also suggested to differ from mesial and lateral groups with regard to coherence of SEEG data.^36^

There is no selective surgery which could show that seizures with almost simultaneous involvement of temporo‐mesial and temporo‐lateral regions have outcomes different from seizures with origin from other temporal subregions; normally patients undergo anterior temporal lobe resections including the amygdala and hippocampus, which provides evidence that the epileptogenic zone was located in the temporal lobe but does not allow to differentiate between temporo‐lateral, temporo‐mesial, temporo‐polar, or temporo‐basal regions when considering the surgical outcome. Thus, the definition of the entity as well as its ascertainment is based on intracranial EEG recordings only.

Using the keywords as given in the Methods section, only two publications were identified which reported semiology in sufficient granularity to be analyzed for this review (Tables 4 and 5).

**TABLE 4:** Studies identified with the search strategy reporting detailed semiology from the “medio‐lateral” temporal regions.

# study	# patients	Adult (A), children (P)	% MRI positive	% SEEG	% operated	% class I ≥1 year	Confidence in the EZ (% very high/high/moderate/low)
1	33/50	50A^a^	Un	30	85	54	0% very high, 30% high, 14% moderate, 56% low
2	18/55	55A	72.3	100	83.3	66.7	67% very high, 33% moderate

*Note*: Confidence in the epileptogenic zone localized “medio‐laterally” was only based on orthogonal intracranial SEEG recordings showing a latency of involvement of 0–3 s between medial and lateral cortex. Un, unknown. Study 1: Chassoux et al.^37^; 2: Maillard et al.^38^

^a^ Age: 14–50 years, otherwise not specified.

**TABLE 5:** Summary of evidence for early “medio‐lateral” temporal involvement.

	# patients	% adults	% MRI positive	% SEEG	% operated	% class I ≥1 year	High or very high confidence in the EZ
Min.	18	100	Un	30	83	54	0
Max.	33	100	72.3	100	85	67	67
Median	25	100	–	65	84	60	33.5
Total	51	100	Un	55%	84%	58%^a^	20%

*Note*: There is no specific surgical approach for this entity so that evidence is based on SEEG findings only.

^a^ Percentage of operated patients, equivalent to 49% of the total group.

Chassoux et al.^37^ reported the semiology of 33 patients with “medio‐lateral” seizure involvement and found frequent oro‐alimentary automatisms, salivation, motor signs (e.g., dystonic posturing and head deviation) as well as transition to bilateral tonic–clonic seizures; the comparatory groups of temporo‐medial and bilateral temporal onset seizures were, however, small, comprising 13 patients with temporo‐medial and four patients with bitemporal seizure generation.

In the most detailed semiological study by Maillard et al.^38^ which included 18 patients with “medio‐lateral” TLE, semiology with early involvement of both median and lateral temporal lobe was intermediate between TLE with medial and lateral seizure origin.

Thus, the most common focal aware epigastric sensation reported, “dreamy states” and oro‐alimentary automatisms as well as a long seizure duration were shared with medial seizure generation, and initial loss of contact with lateral seizure generation. The frequency of occurrence of fear, auditory, gustatory, and sensory hallucinations as well as vertigo was intermediate between medial and lateral onset. Most semiological differences between “medio‐lateral” seizure generation did not reach statistical significance, with the possible exception of more frequent early automatisms and vocalization/verbal automatisms in seizures with rapid medial and lateral involvement (Table 6).

**TABLE 6:** Anatomico‐clinical correlations in seizures with rapid medial and lateral cortical involvement.

Medio‐lateral TL seizures	% reported (min–max)	More at onset or during propagation	Overall grade level (high, moderate, low) that the sign/symptom is associated with the brain region
Early automatisms	56 (1 study)	Early (first half of the seizures)	Moderate
Early vocalization	14 (0–38)	At onset	Moderate
Viscerosensory aura	73 (50–85)	Early	Low
Sensory hallucinations	33 (33–33)	Early	Low
…			

## 4 DISCUSSION

Any attempt to correlate semiology and epileptogenic zone in the brain suffers from the general problem that less than 10% of the cerebral cortex is “eloquent” in the sense that local electrical stimulation induces signs or symptoms without afterdischarges, and similarly localized epileptic ictal patterns will remain asymptomatic without propagation. This limits any expectation to find close associations of isolated clinical manifestations with a predefined small brain volume, unless it is one of the eloquent areas. In the temporal lobe, eloquent regions in this sense are limited to the Heschl gyrus and auditory belt, the language‐dominant temporobasal and Wernicke language areas and possibly the entorhinal cortex.

When studying seizures arising from the temporal pole, another difficulty arises from the technical aspect that several centers make use of depth electrode implantations using a lateral orthogonal approach. This precludes to reach the rostral top of the temporal lobe, thus recordings rather from the border regions between temporal pole or/and main temporal lobe or even from the level close to the amygdala. Similarly, subdural evaluations of the temporal pole pose particular challenges.^33^, ^39^ Best evidence thus derives from patients with very localized brain lesions and seizure freedom after tailored temporo‐polar resections or from centers using oblique electrode trajectories the tip of which actually record from the most anterior 1.5 cm of the temporal lobe. SEEG recordings and/or very circumscribed resections of temporopolar encephaloceles, which can occur congenitally, posttraumatically, and due to idiopathic intracranial hypertension, have recently provided particularly detailed information on electroclinical manifestations which may arise from this regions.

Another problem with available data is that some centers performed standard anterior temporal lobe resections even though they found evidence from intracranial recordings for a subregion of the temporal lobe as the seizure onset zone. This precludes obtaining very high evidence for a localization in the temporal pole (as in other temporal subregions) as analyzed for this systematic review.

Even if patient numbers are low for temporo‐polar epileptogenic zones, some SEEG recordings (e.g.,^34^) very precisely show that the onset of seizure symptomatology co‐occurs with propagation of ictal activity to often distant regions, and that the particular semiology of the seizure is closely related to the topography of propagation rather than to the region of seizure onset. Given the wide connectivity of the temporal pole including frontobasal and frontolateral cortex, insula and temporal regions, the interindividual variability of semiologies reported with seizures of temporo‐polar origin and the low percentage of individual semiological symptoms and signs underlines that connectivity to symptomatogenic regions determines semiology and not the predefined epileptogenic region. This also correlates with one very detailed analyses of polar stimulation results.^40^ There is thus no specific and not even a frequent semiological sign the occurrence of which can be attributed to the temporal pole. Hyperkinetic semiology, as emphasized by Wang et al.,^32^ may also occur with propagation of ictal discharges originating from the temporal pole rather than with primary frontal lobe involvement. Studies on differential connectivity arising from temporo‐polar subregions, for example, using functional tractography,^41^ will thus be needed in order to better understand the individual patients' semiology.

“Medio‐lateral” or “latero‐medial” seizures comprise a subgroup of seizures with has been separated out by the Marseille group based on rapid spread of epileptic activity from the lateral to the mesial temporal region. Overall, spread of ictal activity has been reported to be more rapid with origin from neocortical temporal areas than from the hippocampus,^42^, ^43^ which may suggest that rapid latero‐mesial spread might occur more frequently than contrariwise; this, however, cannot be inferred from the two studies identified on this topic.

Not surprisingly, semiologies of patients with rapid spread within the temporal lobe to a great degree overlap with seizures with slower (>3 s) spread of ictal activity between the two regions. The major result of the detailed analysis of Maillard et al.^38^ was that seizures of lateral seizure origin differed in several respects from those of mesial origin, whereas significant differences between these and the “medio‐lateral” group was only found with regard to the timing of onset of automatisms, which was found to be earlier in the “medio‐lateral” group with rapid ictal propagation. Further evidence is needed to see if additional signs like early vocalization which was found in 28% of Maillard's patients really are related to rapid intra‐temporal rapid ictal spread or rather rapid spread also to other brain regions, particularly to the frontal lobe. Furthermore, there is no specific surgery which could potentially show differential outcomes, and patients operated underwent standard anterior temporal lobectomy including hippocampectomy, precluding additional evidence for this temporal subgroup. Present‐day evidence thus does not support a separation of this “medio‐lateral” group from the 1989 ILAE subclassification of mesiotemporal versus temporal neocortical epilepsies.^44^

This systematic review is limited by the search strategy chosen, which may have excluded relevant literature which was not met by the terms selected. Furthermore, the total number of patients included in the identified studies was limited, and data on children are underrepresented. Nevertheless, there was agreement within the subgroups that seizures of temporo‐polar origin are characterized by highly variable patterns of spread, and that seizures involving rapidly mesial and lateral temporal cortex appear to semiologically represent a mixture of characteristics of the more well‐separable seizures of temporo‐mesial and temporo‐lateral onset.

## Supporting information


Data S1.
