# Supplementary material for: Semiology of seizures with temporo‐polar or “medio‐lateral” temporal origin: A systematic review
Source: Epileptic Disord. 2025 Mar 12;27(2):187–95. doi: 10.1002/epd2.20329 (PMC12065117; doi:10.1002/epd2.20329)
Supplement: Supplementary file 1 — Data S1. [file EPD2-27-187-s001.docx]

**TEST YOURSELF**

**Answers:**

1. D.

2. C.

3. A.
